# Geographic variation of reproductive traits and competition for pollinators in a bird‐pollinated plant

**DOI:** 10.1002/ece3.5457

**Published:** 2019-08-20

**Authors:** Genevieve L. Theron, Caroli de Waal, Spencer C. H. Barrett, Bruce Anderson

**Affiliations:** ^1^ Department of Botany and Zoology Stellenbosch University Matieland South Africa; ^2^ Department of Ecology and Evolutionary Biology University of Toronto Toronto ON Canada

**Keywords:** *Babiana ringens*, bird pollination, Cape floristic region, ecotype, floral evolution, geographic variation, interspecific competition, pollen limitation, self‐pollination

## Abstract

Geographic variation in the reproductive traits of animal‐pollinated plants can be shaped by spatially variable selection imposed by differences in the local pollination environment. We investigated this process in *Babiana ringens* (Iridaceae), an enigmatic species from the Western Cape region of South Africa. *B. ringens* has evolved a specialized perch facilitating cross‐pollination by sunbirds and displays striking geographic variation in perch size and floral traits. Here, we investigate whether this variation can be explained by geographic differences in the pollinator communities. We measured floral and inflorescence traits, and abiotic variables (N, P, C, and rainfall) and made observations of sunbirds in populations spanning the range of *B. ringens*. In each population, we recorded sunbird species identity and measured visitation rates, interfloral pollen transfer, and whether the seed set of flowers was pollen limited. To evaluate whether competition from co‐occurring sunbird‐pollinated species might reduce visitation, we quantified nectar rewards in *B. ringens* and of other co‐flowering bird‐pollinated species in local communities in which populations occurred. Variation in abiotic variables was not associated with geographical variation of traits in *B. ringens*. Malachite sunbirds were the dominant visitor (97% of visits) and populations with larger‐sized traits exhibited higher visitation rates, more between‐flower pollen transfer and set more seed. No sunbirds were observed in four populations, all with smaller‐sized traits. Sunbird visitation to *B. ringens* was not associated with local sunbird activity in communities, but sunbird visitation was negatively associated with the amount of *B. ringens* sugar relative to the availability of alternative nectar sources. Our study provides evidence that *B. ringens* populations with larger floral traits are visited more frequently by sunbirds, and we propose that visitation rates to *B. ringens* may be influenced, in part, by competition with other sunbird‐pollinated species.

## Graphical abstract

We demonstrate that geographic variation in floral morphology is correlated with pollinator visitation rate. Pollinator visitation rates appear to be influenced by competition with plants in the surrounding community. This suggests that competition may play a role in shaping floral evolution.

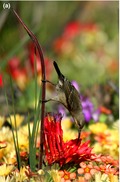

## 1 INTRODUCTION

Much of the striking floral variation within and among angiosperms has been attributed to evolutionary responses to variation in the pollinator environment (Johnson, 2010; Kay & Sargent, 2009). Geographic variation in the pollinator landscape can potentially drive mosaics in selection regimes and floral phenotype (Herrera, Castellanos, & Medrano, 2006; Newman, Manning, & Anderson, 2015; Paudel et al., 2016). For example, differences in the relative abundance of pollinators (Anderson, Alexandersson, & Johnson, 2010; Boberg et al., 2014; van der Niet, Pirie, Shuttleworth, Johnson, & Midgley, 2014), as well as morphology (Anderson & Johnson, 2008) and preference differences in a single pollinator species (Newman, Anderson, & Johnson, 2012) may select for distinct floral ecotypes adapted to different pollinators across the geographical range of a species. Plant responses to mosaics in the quantity and quality of pollinators may include morphological adaptations to the composition of different pollinator communities (e.g., Anderson, Ros, Wiese, & Ellis, 2014; Newman et al., 2015). In addition, evolutionary responses may include changes in mating system associated with variation in the abundance of pollinators (i.e., degree of pollinator limitation; Barrett & Husband, 1990; Eckert, Samis, & Dart, 2006; Lloyd, 1979), although other adaptive responses can also occur (Harder & Aizen, 2010).

Insufficient pollination resulting in reduced seed set can drive shifts from outcrossing to selfing (Darwin, 1876; Goodwillie, Kalisz, & Eckert, 2005; Harder & Barrett, 1996; Jain, 1976; Lloyd, 1992). This transition is associated with changes to a suite of floral traits, including reduced flower size and stigma–anther separation (herkogamy), greater autonomous self‐pollination, less attractive rewards, and lower pollen‐to‐ovule ratios (e.g., Lloyd, 1965; Morgan & Barrett, 1989; Sicard & Lenhard, 2011). Although shifts to selfing have been documented in numerous angiosperm genera through comparative studies (Stebbins, 1974), empirical studies of the ecological context promoting the evolution of selfing are less well‐investigated (Barrett & Harder, 2017; Levin, 2012).

A mechanism proposed for the evolution of increased selfing concerns competition among co‐flowering species for shared pollinators (Campbell, 1985; Levin, 1972). In plant communities where some species are more abundant, or offer superior floral rewards, pollinator service to less rewarding species may be compromised resulting in conditions favoring increased selfing. Although array experiments have demonstrated that interspecific competition between co‐flowering animal‐pollinated species can lower seed production and outcrossing rates (Bell, Karron, & Mitchell, 2005), the extent to which similar processes operate in plant communities with contrasting mating systems is poorly understood (but see Briscoe Runquist, Grossenbacher, Porter, Kay, & Smith, 2016). The relation between pollinator sharing and visitation is further complicated because the co‐occurrence of species may also facilitate increased visitation rates (Moeller, 2004; Thomson, 1978). In addition, the influence of the abiotic environment on resource availability may also indirectly affect visitation rates by changing floral rewards and attractiveness (Carroll, Pallardy, & Galen, 2001; Galen, 2000).

**Figure 1:**
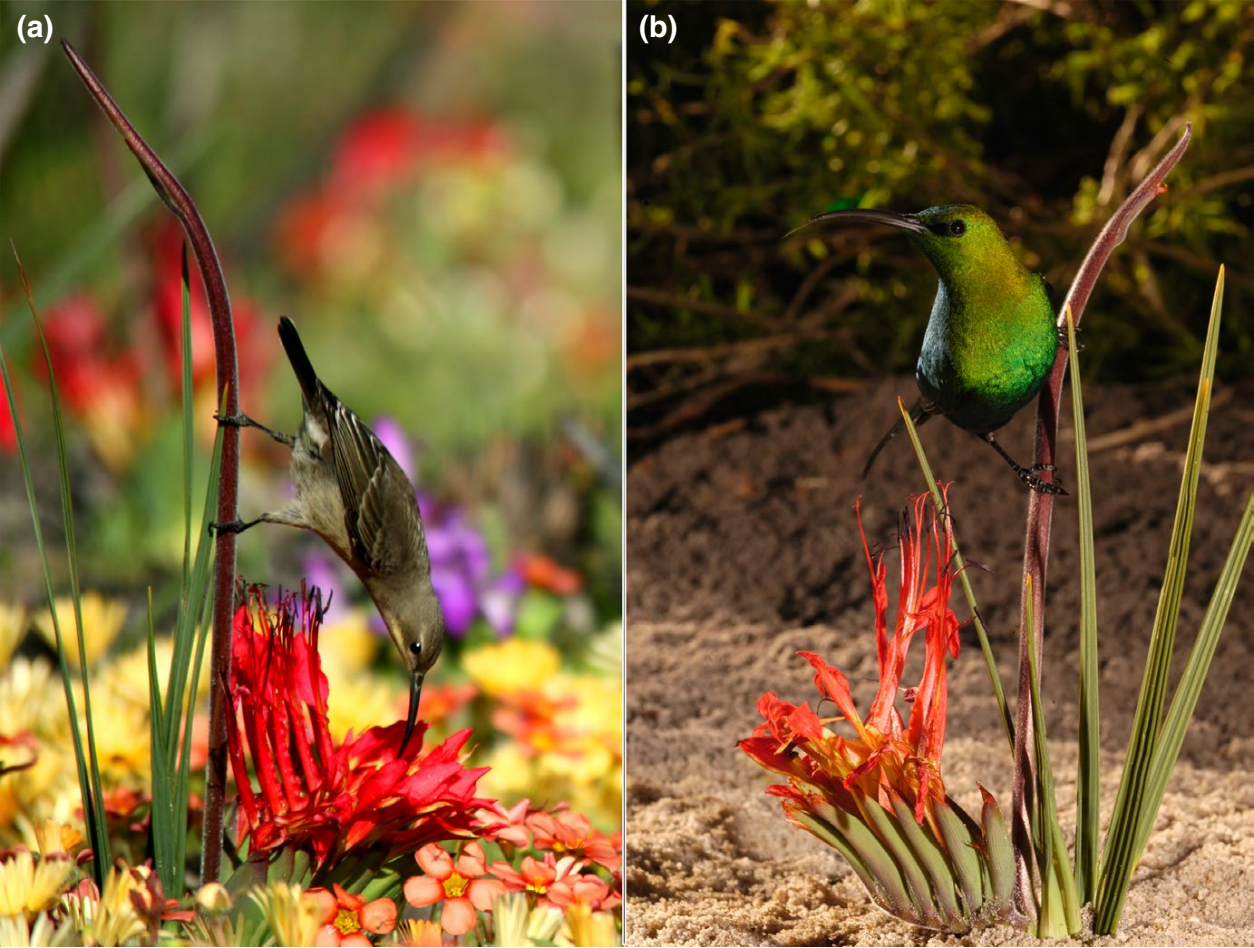
Malachite sunbirds using the perch of *Babiana ringens* while foraging for nectar. (a) Female malachite sunbird leans over the reproductive organs of the flower as it reaches toward the tubular flowers of *B. ringens* situated on the ground. To obtain nectar, the bird must fully insert its bill into the floral tube, at which point the bird's chest will brush against the anthers and stigmas of the inflorescence. (b) Male malachite sunbird uses the perch to gain some elevation from the ground where it is able to call and survey his surroundings, which it does regularly between probing bouts on an inflorescence (also Video S1)

**Figure 2:**
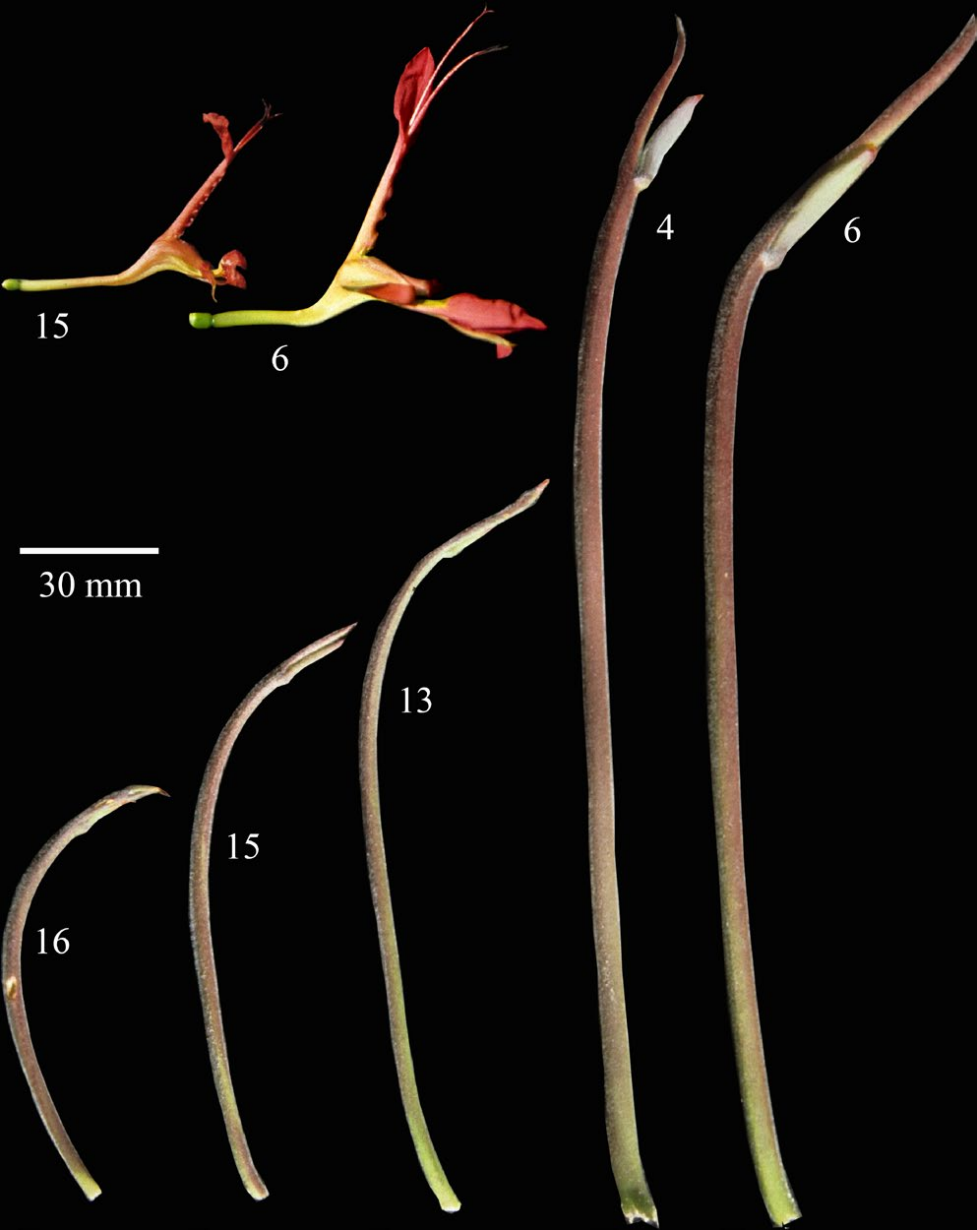
Variation in flower and perch size among populations of *Babiana ringens*. Numbers correspond to those on the map (Figure 3)

*Babiana ringens* (Lin) Ker Gawl (Iridaceae) is a sunbird‐pollinated geophyte, endemic to the Cape floristic region of South Africa (Figure 1). The species possesses striking geographical variation in floral and inflorescence traits (Figure 2) which may be associated with contrasting pollination environments across the Western Cape region (de Waal, Anderson, & Barrett, 2012). The attractive red flowers of *B. ringens* are presented at ground level, an unusual location for a bird‐pollinated species. *Babiana ringens* possesses an unusual naked (flowerless) inflorescence axis which projects above the flowers and functions as a perch enabling sunbirds to probe downwards for nectar (Figure 1; Video S1; Anderson, Cole, & Barrett, 2005; de Waal, Anderson, et al., 2012). Perch removal results in reduced visitation rates by sunbirds and reduced seed set and quality (Anderson et al., 2005). This remarkably specialized adaptation for sunbird pollination occurs in only two species of *Babiana*, the other being the very localized *B. avicularis* Goldblatt & J.C. Manning (de Waal, Anderson, et al., 2012).

de Waal, Anderson, et al. (2012) documented striking variation in *B. ringens* perch size, with population averages ranging from 92.1 to 216.2 mm in the Western Cape (Figure 2). Variation in perch size was positively correlated with flower size, floral‐tube length, and the degree of herkogamy (de Waal, Anderson, et al., 2012), suggesting the correlated evolution of these traits. Geerts and Pauw (2009) reported that sunbird‐pollinated plants in the Cape region could be divided into two distinct guilds: plants with short tubes (15–30 mm), pollinated by short‐billed sunbirds, and plants with long tubes (30–47 mm) pollinated by malachite sunbirds with longer bills. The average floral‐tube length of *B. ringens* populations varies from approximately 25–45 mm (de Waal, Anderson, et al., 2012), spanning the floral‐tube length of both sunbird pollination guilds. Both long‐ and short‐billed sunbirds have been observed visiting *B. ringens,* and one of the goals of this study was to evaluate the hypothesis that variation in floral and inflorescence traits of this species may be shaped by spatially variable selection imposed by the local pollination environment.

Here, we document geographic variation in flower and inflorescence traits of *B. ringens* and investigate whether the observed variation is associated with specific biotic and abiotic factors. Abiotic factors included water and soil nutrients. These could directly influence plant size. Alternatively, they may influence the identity and visitation rates of sunbirds or the availability of nectar resources in *B. ringens* and in other co‐flowering species pollinated by birds. We hypothesized that geographic differences in *B. ringens* floral phenotype are adaptations to short‐ versus long‐billed sunbirds (i.e., variation in visitor composition). Accordingly, we predicted that populations with robust perches, large flowers, and long floral tubes would be visited primarily by large, long‐billed malachite sunbirds, *Nectarinia famosa* Linnaeus. In contrast, we predicted that populations with smaller floral and inflorescence traits would be visited primarily by small sunbird species with short bills such as the southern double‐collared sunbird, *Cinnyris chalybeus* Linnaeus, and orange‐breasted sunbird, *Anthobaphes violacea* Linnaeus.

We established the patterns of variation and covariation among floral and inflorescence traits in a sample of *B. ringens* populations across the geographical range of the species and then asked the following specific questions: (a) Are there associations between *B. ringens* trait variation and abiotic factors among populations? (b) Is geographic variation in reproductive traits associated with visitation by different sunbird species? (c) Is geographical variation in sunbird visitation rates and reproductive traits associated with variation in seed set, pollen limitation, and pollen transfer? (d) Is sunbird activity and relative sugar availability in local bird‐pollinated communities positively associated with visitation rates to *B. ringens*?

## 2 METHODS

### 2.1 Study system

*Babiana ringens* flowers from August to October and has characteristics which fit the sunbird pollination syndrome (Figure 1), including red flowers with long floral tubes, a perch for birds to forage from, large volumes of dilute nectar, and an absence of scent (Anderson et al., 2005; Van der Pijl, 1961). *Babiana ringens* is one of the four sunbird‐pollinated species in this genus of approximately 92 species (Goldblatt & Manning, 2007a, 2007b; de Waal, Anderson, et al., 2012). The striking variation in flower and perch size has led to an east–west division into two subspecies: *B. ringens* subsp. *ringens* Goldblatt & Manning with larger flowers and perches in the west, and *B. ringens* subsp*. australis* Goldblatt & Manning with smaller flowers and perches in the east (Goldblatt & Manning, 2007a).

We observed three sunbird species at our study sites: the malachite, southern double‐collared, and the orange‐breasted sunbird. Each species has been observed visiting *B. ringens* at one or more sites, and all can potentially facilitate pollination. Experimental pollination studies of *B. ringens* indicate that plants are highly self‐compatible, with some capacity for autonomous self‐pollination (de Waal, Anderson, et al., 2012), and marker gene studies have established that the species has a mixed mating system with low to moderate frequencies (*t* = 0.25–0.55) of outcrossing (Anderson et al., 2005; de Waal, Anderson, et al., 2012). Outcrossing rates could only be measured in populations with larger floral and perch phenotypes as no polymorphism at allozyme loci was detected in populations with smaller phenotypes. This probably reflects high selfing rates in these populations (de Waal, Anderson, et al., 2012). Further information on the floral biology of the species is detailed in de Waal, Anderson, et al. (2012) and de Waal, Barrett, and Anderson (2012).

### 2.2 Are there associations between variation in vegetative and floral traits?

During August to October 2009, 2014 and 2015, we made morphological measurements *in situ* from 13 flowering populations of *B. ringens* and obtained additional measurements from another five populations based on herbarium specimens (see Table S1 for localities, measurement, and details of sample sizes). The measurements included perch length, dorsal tepal length, corolla length, length of the longest leaf, and stigma–anther separation. We also recorded the total number of open flowers and buds on each inflorescence (hereafter flowers per inflorescence). In 11 populations, we examined whether perch length in each population covaried with floral trait measurements.

### 2.3 Are there associations between *Babiana ringens* trait variation and abiotic factors?

We collected five soil samples (one kg each) from eight *B. ringens* populations (2, 3, 4, 12, 13, 14, 15, and 16) spanning the phenotypic variation within the species. The soil was removed from a depth of approximately 25 cm (the average depth of *B. ringens* bulbs). We determined total N and C content of soil through total combustion using a Leco Truspec^®^ CN N analyser, by means of the Walkley–Black method (Non‐Affiliated Soil Analysis Work Committee, 1990), and total phosphate was determined by a method adapted from that described by Sommers and Nelson (1972). We extracted phosphate from soil by acid digestion using a 1:1 mixture of 1 N nitric acid and hydrochloric acid at 80°C for 30 min. We then determined the P concentration in the extract with a Varian ICP‐OES optical emission spectrometer. We obtained the historical mean monthly rainfall from the WorldClim data set (Hijmans, Cameron, Parra, Jones, & Jarvis, 2005) with a resolution of 1 km^2^ for each population. The monthly means were summed to give a mean annual rainfall for each of the 11 populations (1, 2, 3, 4, 6, 12, 13, 14, 15, 16, and 18).

To determine whether abiotic factors were associated with size variation in *B. ringens* traits, we conducted regression analyses explaining perch length with % N, % C, phosphate content, and mean annual rainfall as explanatory variables. Because the measurements that we made for inflorescence and floral traits were highly correlated (see Results), we used perch length as a surrogate for corolla length, dorsal tepal length, display size, stigma–anther separation, and the longest leaf. We chose perch length because previous work demonstrated the importance of the perch on visitation and outcrossing rates (see Anderson et al., 2005; de Waal, Anderson, et al., 2012).

### 2.4 Is geographic variation in reproductive traits associated with visitation by different sunbird species?

In 11 populations, we recorded sunbird visitors to *B. ringens* flowers. We chose populations distributed across the entire range of the species, spanning the phenotypic variation in traits of interest. To determine whether trait variation was associated with particular sunbird species, we conducted a total of 60 hr of pollinator observations over 1–5 days per site (mean = 2.9 days), with a minimum of 30 min of observation each day in fine weather (Table S2). We made all observations soon after sunrise at a vantage point which allowed us to simultaneously view the greatest number of plants in a population. At three sites (1, 6, and 18), we used five camera traps (Ltl Acorn^®^ Ltl‐6210MC) to observe plants outside our field of view. In these populations, plants were sparsely distributed and we used cameras to increase the total number of plants observed. A single camera was placed about a meter away from plants and set to record for 60 s when triggered by motion. We only set cameras during periods of human observation in these populations. A visit was recorded when sunbirds landed on a perch and probed a flower for nectar. The mean visitation rate per population was standardized as the number of visits per plant per hour. We then used visitation rate in regressions as the predictor variable of perch length.

### 2.5 Is geographic variation in visitation rates and reproductive traits associated with variation in seed set, pollen limitation, and pollen transfer?

To assess the effect of sunbird visitation on a female fitness component, we determined whether natural (open‐pollinated) seed set varied with bird visitation rate. This was done by regressing seed set with visitation rate across 11 populations. To determine whether seed set among populations was limited by pollen receipt (pollen limitation), we used the following standardized metric: we calculated pollen limitation in each of the populations as pollen limitation = 1 – (unmanipulated/pollen‐supplemented), where 0 indicates no pollen limitation and 1 complete pollen limitation (See Table S1 for sample sizes in each population). We marked unmanipulated buds and these were left undisturbed, whereas receptive stigmas of pollen‐supplemented flowers received pollen from two pollen donors by removing anthers from donor flowers and rubbing them on all stigma lobes of recipient flowers. Donors were located more than two meters from recipients. Using regression, we then determined whether the severity of pollen limited seed set decreased with increasing sunbird visitation rate across populations.

Although bird visitation rates may not affect seed set in species capable of autonomous self‐pollination, high levels of pollinator activity should result in more pollen dispersal among flowers and higher outcrossing rates. To determine whether there was a positive association between visitation rate and pollen movement between flowers, we emasculated flowers in bud prior to anther dehiscence, thus ensuring that seed set could only occur as a result of interfloral pollen transfer. We regressed visitation rates in the 11 populations with an estimate of pollen dispersal between flowers in each population. Between‐flower pollen movement in each population was estimated as seed set of emasculated/pollen‐supplemented treatments, where 0 indicates no interfloral pollen transfer and 1 shows complete interfloral pollen transfer. We conducted all three treatments (unmanipulated, emasculated, and pollen‐supplemented flowers) on each replicate plant to limit the influence of between‐plant habitat and plant condition effects. Although our measure of “interfloral pollen transfer” does not distinguish between outcross and geitonogamous components of pollination, it is probable that outcrossing in a population is positively affected by the amount of pollen dispersed among flowers.

We determined whether there was an association between interfloral pollen transfer in each population and *B. ringens* phenotype, particularly stigma–anther separation. Reduced herkogamy may be expected in populations with low interfloral pollen transfer because reduced herkogamy is known to facilitate autonomous self‐pollination and increase selfing rates (Barrett & Shore, 1987; Brunet & Eckert, 1998; Holtsford & Ellstrand, 1992; Motten & Antonovics, 1992; Takebayashi, Wolf, & Delph, 2006).

### 2.6 Is sunbird activity and relative sugar availability in communities positively associated with visitation rate to *Babiana ringens*?

To determine whether sunbird activity in plant communities was associated with visitation rates to *B. ringens*, we collected additional data on sunbird activity independent of our visitor observations*,* described above. This involved an additional 20.7 hr (mean = 1.9 hr, range = 90 – 300 min per population) of observations of plant communities in which *B. ringens* populations occurred (Table S2). We recorded sunbirds when they were within the immediate observation area where *B. ringens* plants occurred and could be identified accurately (Table S2). If a sunbird perched on a different flowering plant species than *B. ringens* or on substrate, it was considered to be a new record, as it was not possible to keep track of individual sunbirds. We then determined by regression whether there was an association between overall sunbird activity in the community and visitation rates to *B. ringens*.

Because some *B. ringens* populations co‐occurred with other species of bird‐pollinated plants (Table S2), we assessed whether competition for pollinators may play a role in determining visitation rates. In each *B. ringens* population, as described in more detail below, we estimated the amount of sugar produced by *B. ringens*, as well as the amount of sugar produced in the immediate vicinity by other co‐occurring bird‐pollinated species. From these data, we were able to determine the average amount of sugar available per *B. ringens* plant and the total amount of sugar available in *B. ringens* flowers in each population and also in co‐occurring sunbird‐pollinated plants. This enabled us to evaluate whether sunbirds visit *B. ringens* less frequently in populations where there are abundant, alternative nectar sources, and also whether floral trait and perch‐size variation may be associated with variation in nectar rewards.

To calculate the relative nectar availability of *B. ringens* compared to the overall community, we located all flowering *B. ringens* plants in each population and counted the total number of open flowers on each plant. All other bird‐pollinated species within the immediate vicinity of the population were identified based on Van der Pijl's (1961) classification of pollination syndromes, as well as through direct observations of sunbird visitation. We recorded a total of eight co‐flowering bird‐pollinated species among the 11 *B. ringens* populations (see Table S2). We also estimated the number of flowers or inflorescences on all other bird‐pollinated species within the same area as the *B. ringens* population. This was undertaken by counting the number of flowers per inflorescence from five different plants of each species using five inflorescences per plant. From these values, we approximated the total number of open flowers of all bird‐pollinated species within each of our 11 study sites. We counted flowers for all species except members of the Proteaceae, which have inflorescences too large and densely packed with flowers to allow visual counting. For species of Proteaceae, we therefore counted the number of inflorescences per plant.

For a subset of each bird‐pollinated species at each site (see Table S2 for a list of bird‐pollinated species at each site), we measured nectar volumes using a 5‐µl microcapillary tube and concentrations (g sucrose per 100 g solution) using a handheld refractometer (Bellingham and Stanley, UK). We collected flowers for nectar measurements between 06hr00 and 07hr00 to reduce opportunities for birds to consume nectar. We calculated the total amount of sugar per flower by using a combination of nectar volume and concentration values for each flower (see Bolton, Feinsinger, Baker, & Baker, 1979). Where possible, we took measurements from 25 individuals of each species. However, in several populations there were fewer individuals available, and in those cases, we took nectar measurements from all individuals at a site. Overall, we obtained nectar from a mean ± *SE* of 15 ± 4.46 individuals per species. By multiplying the total amount of sugar (mg) per flower by the total number of open flowers per species, we calculated the amount of sugar available for each bird‐pollinated species per site. At each site, we then calculated the total amount of sugar produced by the *B. ringens* population and divided this by the total sugar produced for the entire sunbird‐pollinated community to give a percent value. This value was arcsine square‐root‐transformed and used as the predictor variable to explain sunbird visitation rate using regression.

The relative nectar rewards of *B. ringens* compared to the overall community were highly clumped. Most *B. ringens* populations either produced 100% of the nectar available to birds or a much smaller proportion of the nectar available to birds. Therefore, we analyzed the data categorically. We divided populations into two groups: populations in which *B. ringens* sugar either made up less than or more than 50% of the nectar available to birds. We then performed a Wilcoxon test to determine whether sunbird visitation rates to *B. ringens* were higher in populations with fewer alternative nectar sources. Similarly, we analyzed whether the communities of bird‐pollinated plants found in association with small‐perched (<145 mm) *B. ringens* populations produced more nectar than communities with large‐perched (>175 mm) populations. Because reward size at the plant level may affect the “profitability” of foraging from a plant, we also used regression to determine whether the average perch size of *B. ringens* plants in each population was associated with the average amount of sugar available to pollinators per plant. All statistical analyses in this study were performed using the standard functions in R (R Core Team, 2017).

## 3 RESULTS

### 3.1 Are there associations between variation in vegetative and floral traits?

**Figure 3:**
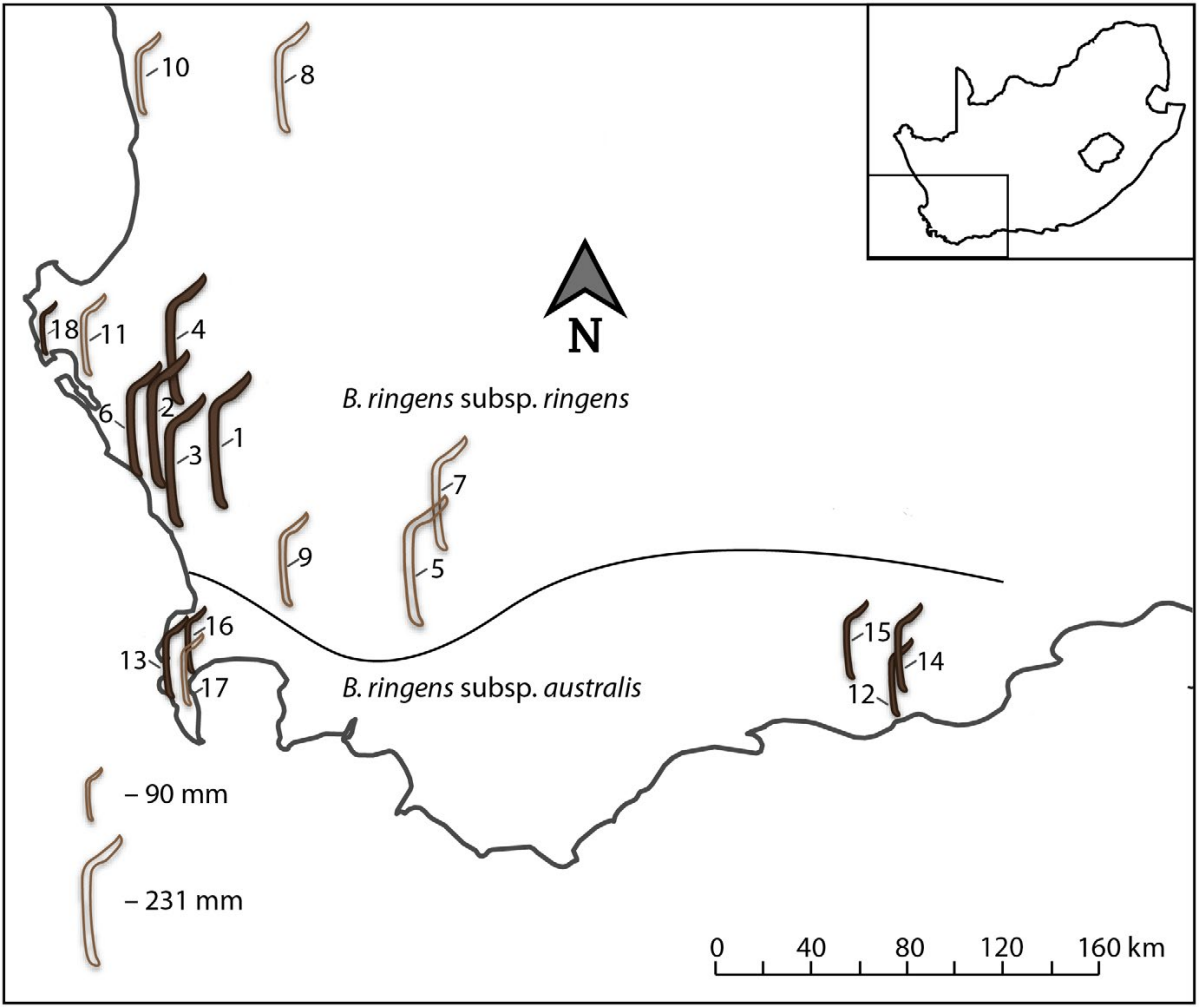
Distribution and perch‐length variation of the two *Babiana* subspecies. The geographic position of each population is depicted by a perch icon, and the mean perch length within a population is proportional to the height of the perch icon. Empty perches depict populations where we only recorded floral/inflorescence trait measurements. Filled in perches depict populations where both measurements and pollinator observations were carried out. GPS coordinates for populations are available in Table S1

Significant morphological variation was evident among populations of *B. ringens* across its geographic range. Perch size varied from 89.5 to 231 mm. Small perches were not restricted to *B. ringens* subsp*. australis* populations and instead occurred at the range extremities with large‐perched populations occupying the more central portions of the range (Figure 3). Perch size was positively correlated to multiple floral traits including dorsal tepal length (*t*_9_ = 7.07, *r* = .92, *p* < .001), floral‐tube length (*t*_9_ = 2.48, *r* = .64, *p* = .0347), stigma–anther separation (*t*_9_ = 3.88, *r* = .79, *p* = .004), and the number of flowers per inflorescence (*t*_9_ = 4.89, *r* = .85, *p* < .001). Perch length was also positively correlated to the length of the longest leaf (*t*_9_ = 2.90, *r* = .70, *p* = .018) but not to the volume of nectar per flower (*t*_9_ = 1.14, *r* = .36, *p* = .282).

### 3.2 Are there associations between *Babiana ringens* trait variation and the abiotic environment?

There were no associations between mean perch length of *B. ringens* populations and average rainfall at each location (*F*_1,9_ = 0.95, *r* ^2^ = .10, *p* = .355), total nitrogen (*F*_1,6_ = 1.18, *r* ^2^ *=* .16, *p* = .319), phosphorus (*F*_1,6_ = 1.44, *r* ^2^ = .19, *p* = .275), or carbon content (*F*_1,6_ = 2.86, *r* ^2^ = .32, *p* = .142) of soil in which plants were growing.

### 3.3 Is geographic variation in reproductive traits associated with visitation by different sunbird species?

**Figure 4:**
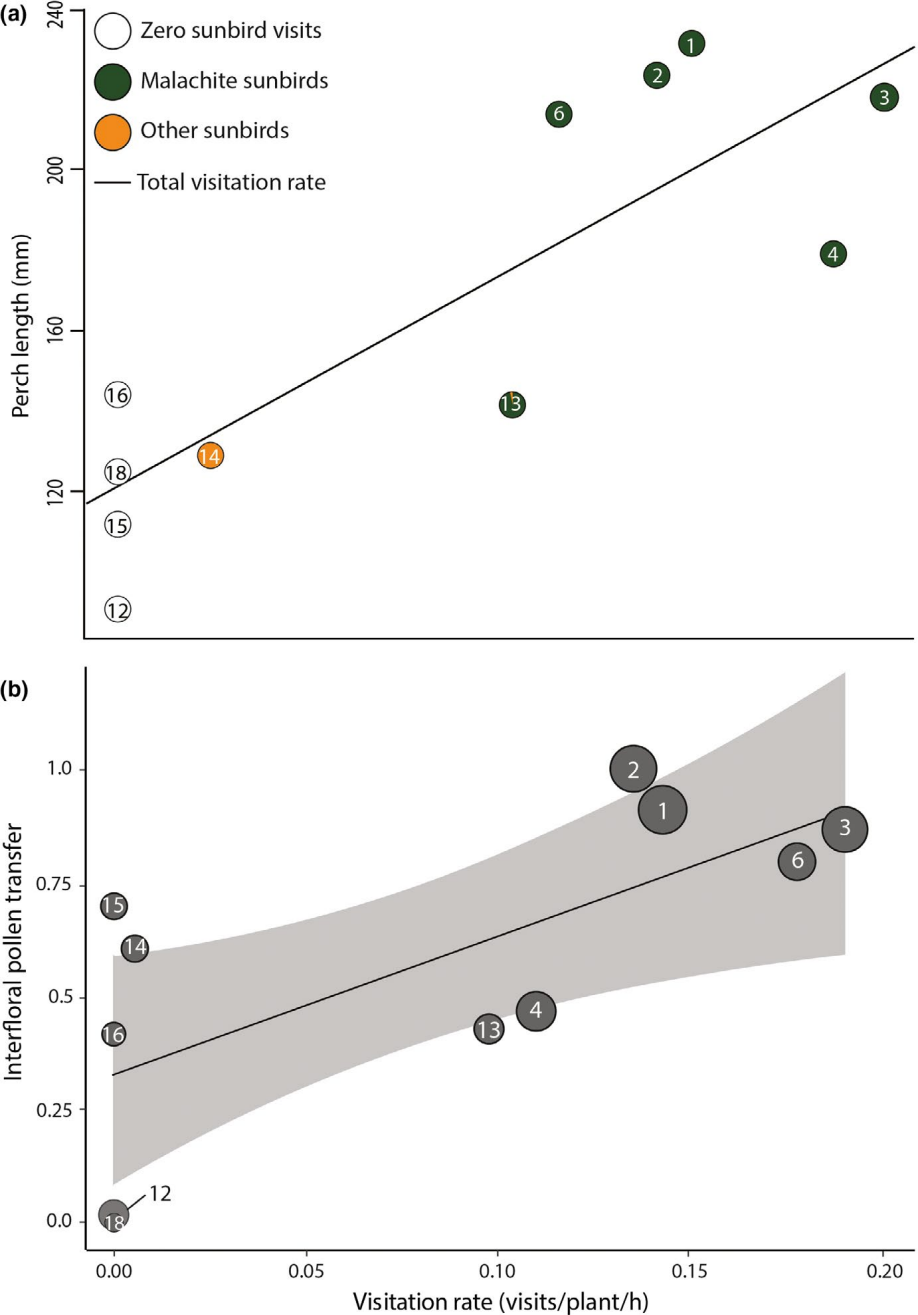
Relationship between visitation rate (visits/plant/hour) by sunbirds and (a) perch length (mm) (*F*_1,9_ = 22.57, *r* ^2^ = .715, *p* = .001). Population 13 was visited by two groups of sunbirds, and the colors within the symbol represent the percent contribution of each sunbird group (small sunbird species and large malachite sunbirds) to the total visitation rate; (b) interfloral pollen transfer (*F*_1,9_ = 8.40, *r* ^2^ = .48, *p* = .018) in 11 populations of *B. ringens*. The shaded area around the trend line indicates the 95% confidence interval, and the circle size represents the relative perch length in each population. Numbering follows those in Figure 3 and Table S1

During field observations of the 11 populations, a total of 110 sunbird visits were recorded to inflorescences of *B. ringens*. Of these, 97.27% were by malachite sunbirds and the remaining visits were from two smaller sunbird species with short bills. In all five populations with larger floral and inflorescence traits, only malachite sunbirds were observed visiting flowers (Figure 4a). We observed no sunbird visitors in most (4/6) populations with small floral and inflorescence traits (populations 12, 15, 16, and 18). Sunbird visitors were, however, observed in two populations with small floral and inflorescence traits (populations 13 and 14). The majority of visitors to population 13 were malachite sunbirds, although one visit by an orange‐breasted sunbird was observed (Figure 4a, Table S2). Two visits from southern double‐collared sunbirds were observed in population 14. Thus, there was no strong evidence to support the hypothesis that morphological differentiation in *B. ringens* is associated with visitation by contrasting sunbird species.

### 3.4 Is geographic variation in visitation rates and reproductive traits associated with variation in seed set, pollen limitation, and pollen transfer?

**Figure 5:**
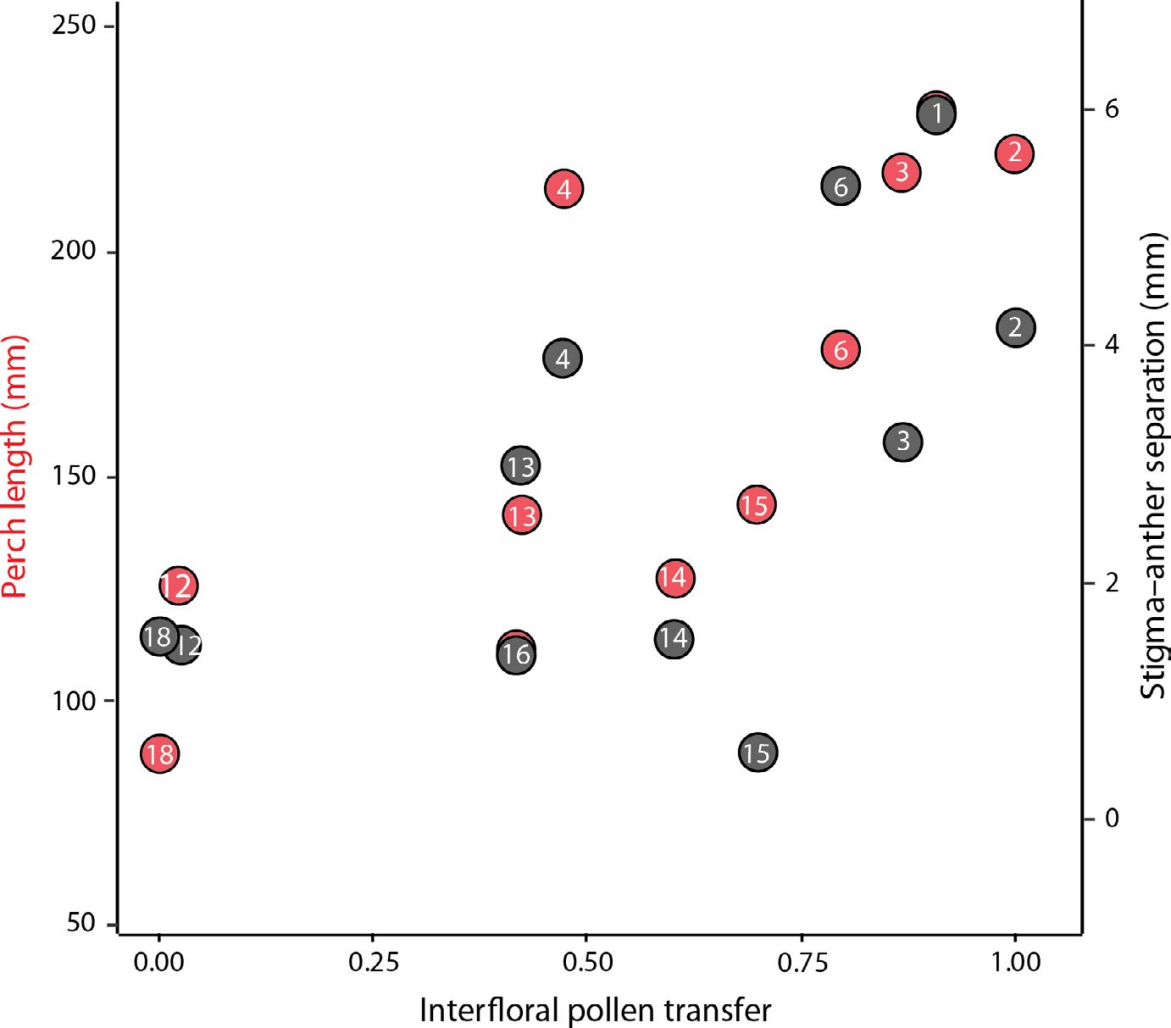
Relationship between interfloral pollen transfer and mean perch length (mm) (*t*_9_ = 3.75, *r* = .781, *p* = .004, red) and stigma–anther separation (*t*_9_ = 2.20, *r = *.592, *p* = .055, black) across 11 populations. Numbering follows those in Figure 3 and Table S1

Estimates of visitation rates in populations had no significant effect on open‐pollinated seed set per flower (*F*_1,9_ = 0.016, *r* ^2^ = .002, *p* = .904). The amount of pollen limitation was low for most *B. ringens* populations (mean  = .07, range = 0 ‐ .37) and was not affected by sunbird visitation rate (*F*_1,9_ = 0.70, *r* ^2^ = .072, *p* = .424). However, seed set per flower resulting from pollen transfer between flowers was positively associated with higher sunbird visitation rates (*F*_1,9_ = 8.40, *r* ^2^ = .48, *p* = .018, Figure 4b). Sunbird visitation was also significantly higher in populations with larger perches (*t*_9_ = 4.75, *r* = .846, *p* = .001), and higher rates of interfloral pollen transfer were also positively associated with larger perches (*t*_9_ = 3.75, *r* = .781, *p* = .004) and greater stigma–anther separation (*t*_9_ = 2.20, *r* = .592, *p* = .055, Figure 5).

### 3.5 Is sunbird activity and relative sugar availability in communities positively associated with visitation rate to *Babiana ringens*?

Observations of sunbirds visiting communities of bird‐pollinated plants at *B. ringens* populations revealed that malachite sunbirds were present at all 11 sites although in four populations they were not observed visiting *B. ringens*. Site 2 had the lowest sunbird activity while site 6 had the highest, with 1.0 and 16.6 sightings per hour, respectively. Despite this variation in sunbird activity among sites, there was no evidence that sunbird activity in the community was significantly associated with sunbird visitation to *B. ringens* (*F*_1,9_ = 0.140, *r* ^2^ = .154, *p* = .717). Similarly, the activity in communities with only malachite sunbirds was not associated with their visitation rate to *B. ringens* (*F*_1,9_ = 0.004, *r* ^2^ < .001, *p* = .953).

**Figure 6:**
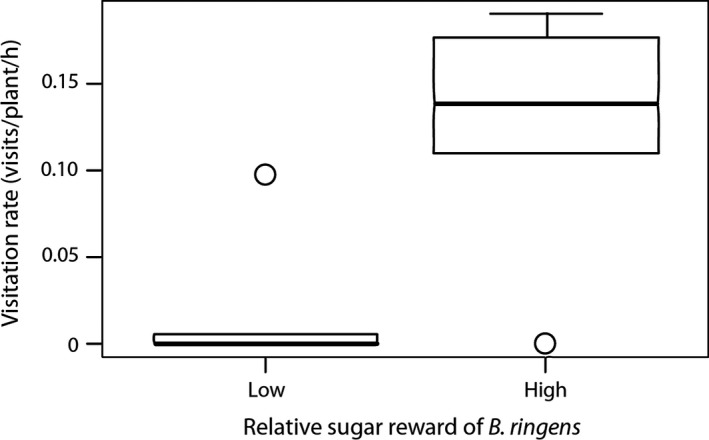
Differences in sunbird visitation rates to *Babiana ringens* in populations in which most of the sugar available to sunbirds was produced by *B. ringens* (high) versus populations in which most of the sugar was produced by other bird‐pollinated species (low) (*W*_9_ = 26.5; *p* = .013). The box margins indicate the upper and lower quartiles, the midline indicates the median, and the whiskers indicate the highest and lowest values excluding outliers (dots)

In eight of the 11 *B. ringens* populations, other bird‐pollinated species were co‐flowering to varying degrees of abundance, and these species received many sunbird visits during our observation periods (Table S2). *Babiana ringens* populations in communities with relatively low quantities of alternative sugar sources for sunbirds received significantly higher visitation rates than populations in communities with relatively larger quantities of alternative sugar sources (*F*_1,9_ = 5.534, *r* ^2^ = .381, *p* = .04, *W*_9_ = 26.50; *p* = .013, Figure 6). The total standing crop of sugar in *B. ringens* populations with smaller perches did not differ from the total standing crops of sugar in populations with larger perches (*W*_9_ = 3.00, *p* = .082). However, at the plant level, total sugar per plant tended to be lower in populations with small perches than in plants from populations with large perches (*F*_1,9_ = 7.96, *r* ^2^ = .469, *p* = .020). Populations with smaller perches were associated with communities of other bird‐pollinated plants that had significantly larger standing crops of sugar than populations with larger perches (*W*_9_ = 25.00, *p* = .028, Figure S1).

## 4 DISCUSSION

Geographic variation in floral and inflorescence traits of *B. ringens* is associated with variation in the visitation rates of pollinating sunbirds. However, there was no evidence that morphological variation among populations of *B. ringens* was associated with visitation by contrasting sunbird species (i.e., large vs. small sunbirds), as is typically predicted in models of pollinator‐driven divergence through pollinator shifts (e.g., Grant & Grant, 1965; Johnson, 2010). Instead, populations of *B. ringens* with larger flowers and perches had significantly higher overall visitation rates by sunbirds than populations with smaller‐sized traits. Below, we discuss how geographic variation in pollinator visitation may influence the reproductive success of *B. ringens* populations, how reproductive‐trait variation could have arisen, and why pollinator visitation rates vary so greatly among populations.

### 4.1 How might variation in pollinator visitation rates affect maternal fitness components?

Variation in visitation rates to animal‐pollinated plants can affect maternal fitness components through two main avenues: seed quantity and quality. Insufficient pollinator service can reduce the quantity of seeds produced and may result in selection on floral traits. For example, low visitation can select for reproductive assurance to maintain maternal fertility resulting in reduced flower size and herkogamy (Barrett & Harder, 2017; Eckert et al., 2006; Lloyd, 1965; Opedal, 2018; Sicard & Lenhard, 2011). Although we found no evidence for a relation between pollinator visitation rate and seed production in our study, this is probably because of the capacity for autonomous seed production in *B. ringens* (de Waal, Anderson, et al., 2012, see Figure 5), which would have maintained some degree of maternal fertility despite limited sunbird visitation. The occurrence of reproductive assurance by autonomous self‐pollination in species with specialized pollination systems, such as *B. ringens*, appears to be more widespread among animal‐pollinated species than is often appreciated (Fenster & Marten‐Rodriguez, 2007). *Babiana ringens* relies solely on sunbirds for cross‐pollination making populations especially vulnerable to unreliable pollinator service, and this probably explains why populations have evolved the capacity for autonomous self‐pollination.

Visitation rates may affect female fitness as a result of the quality of seeds that a plant produces and if plants are capable of autonomous selfing, as in *B. ringens*, low visitation rates can potentially lead to increased selfing rates. Indirect evidence for this hypothesis was that sunbird visitation rates were positively associated with the amount of pollen transferred between flowers. This should have promoted more outcrossing and higher seed quality. However, our measurements of pollen movement could not distinguish between transfer of pollen between flowers on the same plant (geitonogamous self‐pollination) and pollen dispersal between plants (outcrossing) so this inference is tentative. Further work using genetic markers would be required to determine the quantitative relations between visitation rate and outcrossing rate and how much selfing arises from within‐flower and between‐flower self‐pollination (see Eckert, 2002; Hu, Barrett, Zhang, & Liao, 2005; Schoen & Lloyd, 1992). An earlier marker gene study of *B. ringens* established a direct link between visitation rates and mating patterns by demonstrating that perch removal reduced sunbird visitation rates and increased selfing compared to plants with intact perches. However, the relevance of this manipulation for inferring relations between visitation rates and outcrossing should be treated with caution. Perch removal changed the physical orientation of visiting sunbirds while probing flowers of *B. ringens,* and this change to plant phenotype may not be biologically relevant to contemporary interactions between sunbirds and unmanipulated *B. ringens* plants.

### 4.2 Why do *Babiana ringens* floral and inflorescence phenotypes vary geographically?

Pollinator visitation rates and the amount of interfloral pollen transfer were both correlated with geographic variation of *B. ringens* phenotypes. Low visitation rates and limited interfloral pollen transfer were associated with populations that had smaller flowers, inflorescences, and less nectar per plant. This suggests that sunbird visitation rates may be a factor contributing to geographic variation in *B. ringens* floral and inflorescence traits. This could occur if visitation rates are correlated with mating patterns. Earlier work has demonstrated a lack of heterozygosity at allozyme loci and dissolution of the bird perch in populations with smaller flowers and perches (de Waal, Anderson, et al., 2012). Low allozyme heterozygosity is generally associated with high rates of selfing (Hamrick & Godt, 1996), and this may have led to relaxed selection on perch characteristics (i.e., dissolution of perches) in these populations. Furthermore, investigations of related outcrossers and selfers commonly report that high selfing rates are associated with reduced vegetative, floral, and inflorescence traits among populations (Lloyd, 1965; Vallejo‐Marín & Barrett, 2009), between related species (Armbruster et al., 2002; Goodwillie, 1999; Wyatt, 1984), and in phylogenies (Goodwillie et al., 2010). Reductions in floral traits (e.g., flower size, reward size, and stigma–anther separation) are often interpreted as adaptive responses to the evolution of selfing (Brunet & Eckert, 1998; Charlesworth & Charlesworth, 1981; Charnov, 1982) resulting from chronic pollinator limitation (e.g., Lloyd, 1965; Morgan & Barrett, 1989; Sicard & Lenhard, 2011).

An additional complexity in efforts to explain the relations between reproductive variation, sunbird visitation rates, and interfloral pollen transfer in *B. ringens* is that sunbirds may simply have a preference for larger *B. ringens* plants. As a consequence, individuals in populations with smaller plants may be visited less frequently. Several studies have found that plant phenotype is highly predictive of pollination visitation networks (Biddick & Burns, 2018; Klumpers, Stang, & Klinkhamer, 2019). For example, Biddick and Burns (2018) demonstrated that plant species with large flowers received higher visitation rates from birds than plants with smaller flowers. Although this suggests that selection by birds may favor the evolution of larger‐flowered individuals, it does not explain why so much size variation exists among *B. ringens* populations when sunbirds are abundant everywhere. Determining whether the patterns of sunbird visitation in *B. ringens* populations are a cause or a consequence of geographical variation in reproductive traits highlights the difficulties of correlational studies such as ours. Studies of phenotypic selection within populations or reciprocal transplant experiments between populations with divergent phenotypes are often employed in conjunction with correlative studies on geographic variation (see review by van der Niet, Peakall, & Johnson, 2014). For example, patterns of geographic covariation in floral‐tube and pollinator proboscis length have often been explained by experiments demonstrating pollinator selection on floral‐tube length (e.g., Anderson et al., 2010; Paudel et al., 2016). Future work on *B. ringens* investigating whether sunbird pollinators have preferences for plants with larger floral traits within populations would be valuable.

Another plausible explanation for geographic variation in *B. ringens* phenotype is that it is driven by features of the abiotic environment, either as plastic responses or as evolved responses to local conditions. However, we found no relations between the most limiting resources (N, P, rainfall) in the Cape (see Cramer, West, Power, Skelton, & Stock, 2014) and perch size. It is of course still possible that unmeasured abiotic factors may contribute to phenotypic variation. In addition, it is also unclear to what extent developmental constraints associated with allometry might also limit the independent evolution of different morphological traits (e.g., Niklas, 1994; West, Brown, & Enquist, 1999; but see Biddick, Hutton, & Burns, 2018). Common garden studies would be useful to determine how much of the observed variation has a heritable basis, regardless of the specific ecological and evolutionary processes that may be responsible for the origin and maintenance of geographical differentiation.

### 4.3 Why do pollinator visitation rates to *Babiana ringens* populations vary?

Our study demonstrated that pollinator visitation rates in *B. ringens* populations with smaller floral and inflorescence traits were not associated with lower sunbird activity in the surrounding plant community. Rather, the variation appears to be related to differences in the attractiveness of *B. ringens* plants. First, plants in populations with smaller phenotypes produce reduced nectar rewards for birds and are likely to be less attractive in comparison with other bird‐pollinated plants in the community. Second, the abundance of alternative nectar sources was also associated with visitation rates to *B. ringens,* suggesting a competitive role played by the rest of the bird‐pollinated community. In support of this hypothesis, five of the six populations with smaller flowers and inflorescences coexisted with communities of other sunbird‐pollinated species and pollinators were frequently observed visiting for nectar. In contrast, only one of the five populations with larger flowers and inflorescences had co‐flowering bird‐pollinated plants and these were not especially abundant. In most populations with larger reproductive phenotypes, sunbirds were obliged to visit *B. ringens* for nectar because there were either few or no other nectar sources in the community.

These differences in community context suggest that variation in sunbird visitation rates among populations of *B. ringens* may be determined, in part, by interspecific competition for pollinators. In plant communities of the Western Cape, *B. ringens* may be an especially poor competitor for sunbird pollinators for two main reasons. First, inflorescences of this species are presented at ground level and this particular deployment of flowers is unusual among sunbird‐pollinated plants; notable exceptions are several species of root parasites which produce copious nectar (Hobbhahn & Johnson, 2015; Quintero, Genzoni, Mann, Nuttman, & Anderson, 2016; Turner & Midgley, 2016). Second, in comparison with many of the other sunbird‐pollinated plants that we recorded coexisting with *B. ringens,* the total amount of nectar offered by *B. ringens* is relatively small. *Babiana ringens* is a diminutive geophyte that usually produces a single inflorescence with a relatively small daily display size comprised of less than half a dozen open flowers. In contrast, many competing sunbird‐pollinated species are shrubs with much larger floral displays (e.g., *Erica*, *Leucospermum*, *Mimetes*, and *Salvia*). The relatively small and less elevated floral displays of *B. ringens* are probably the most important reasons for the low visitation rates of sunbirds to this species when taller and more rewarding species are present in the local vicinity. Therefore, reductions in sunbird visitation resulting from competition with other sunbird‐pollinated species are potentially an important factor influencing the prevalence of selfing and floral evolution in *B. ringens*. Although the idea that competition among plants for pollinators could provide a stimulus for the evolution of selfing was originally proposed by Levin (1972) over 45 years ago, it has received surprisingly limited empirical support from studies of natural populations. This general lack of evidence is probably because of the challenge of experimentally disentangling the numerous community‐level influences on pollinator visitation to animal‐pollinated species and the diverse ecological factors affecting plant mating.

## CONFLICT OF INTEREST

None declared.

## AUTHOR CONTRIBUTIONS

Genevieve Theron collected, analyzed, and wrote much of the manuscript, Bruce Anderson conceived of the idea, collected data, and wrote parts of the manuscript, Spencer Barrett helped with writing and editing, and Caroli de Waal helped with data collection.

## Data Availability

We have archived our data on Dryad: https://doi.org/10.5061/dryad.7hp0168 (Theron et al., 2019).
