# Supplementary figures and images for: Geographic variation of reproductive traits and competition for pollinators in a bird‐pollinated plant
Source: Ecol Evol. 2019 Aug 20;9(18):10122–34. doi: 10.1002/ece3.5457 (PMC6816071; doi:10.1002/ece3.5457)

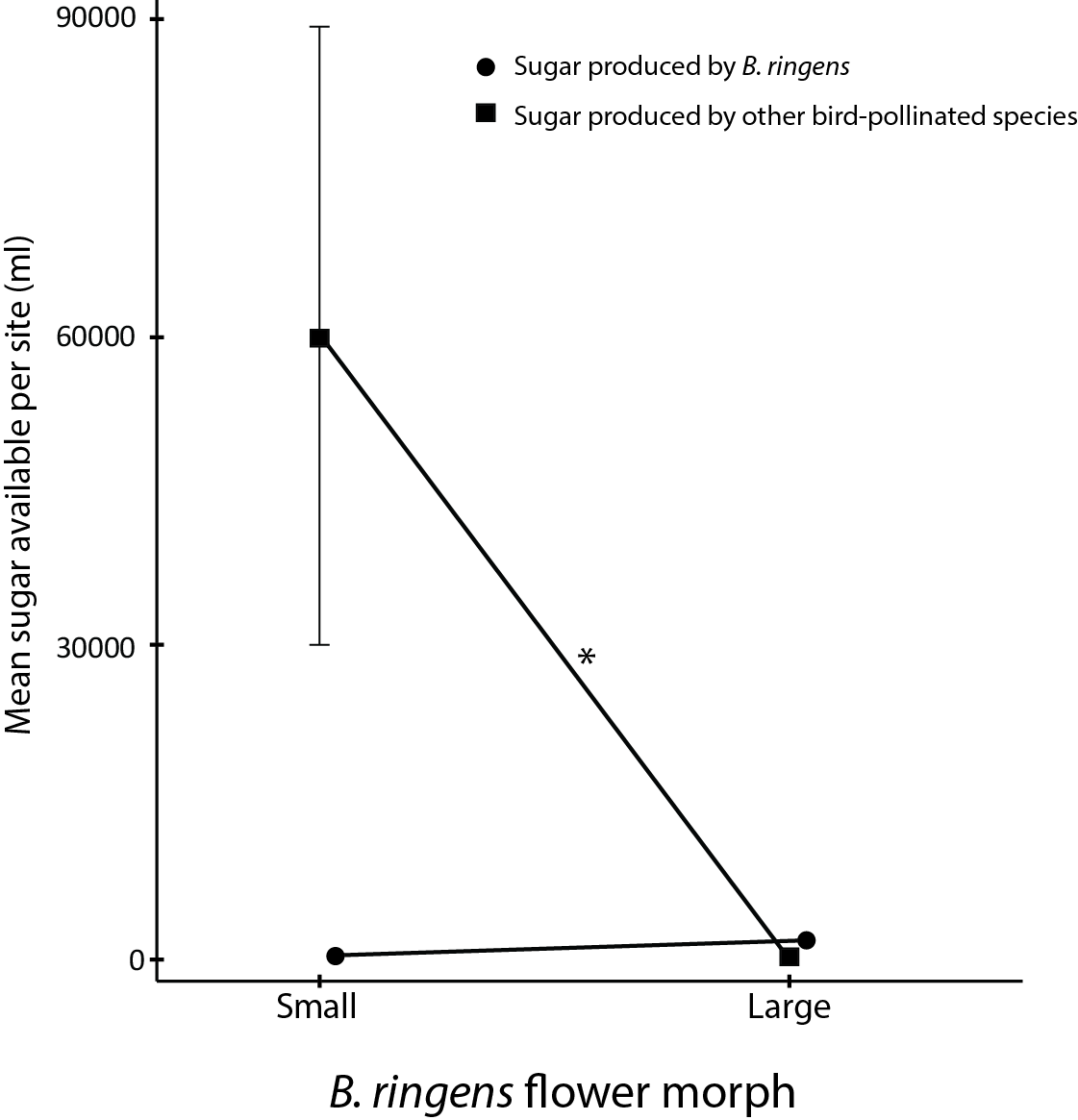

Supplement: Supplementary file 1 [file ECE3-9-10122-s001.png]
