# Supplementary material for: Geographic variation of reproductive traits and competition for pollinators in a bird‐pollinated plant
Source: Ecol Evol. 2019 Aug 20;9(18):10122–34. doi: 10.1002/ece3.5457 (PMC6816071; doi:10.1002/ece3.5457)
Supplement: Supplementary file 2 [file ECE3-9-10122-s002.docx]

**Table S1** Locality information and population means ± standard error, with sample size in brackets, of floral measurements and experimental treatments.

| **Study sites** | | | **Floral Measurements (mm)** | | | | | | | | **Numbers of seeds after floral Manipulations** | | |
| --- | --- | --- | --- | --- | --- | --- | --- | --- | --- | --- | --- | --- | --- |
| **Map #** | **Locality** | **GPS Coordinates** | **Perch length** | **Nectar volume** | **Nectar concentration** | **Longest leaf** | **Flowers per inflorescence** | **Stigma-anther separation** | **Dorsal tepal length** | **Floral tube length** | **Emasculated** | **Unmanipulated** | **Supplemented** |
| 1 | Riverlands Nature Reserve | S 33° 30' 28.4“ E 18° 33' 13.5" | 231.03 ± 7.66 (15) | 35.10 ± 9.93 (5) | 28.50 ± 3.80 (5) | 191.32 ± 10.98 (17) | 12.67 ± 0.69 (18) | 5.94 ± 0.90 (17) | 53.79 ± 0.69 (18) | 43.13 ± 1.0 (18) | 12.43 ± 3.02 (7) | 13.78 ± 1.76 (9) | 13.71 ± 2.16 (7) |
| 2 | Rondeberg Private Nature Reserve | S 33° 24’ 56.5” E 018° 18’ 14.0” | 221.83 ± 6.11 (46) | 18.10 ± 1.68 (25) | 23.44 ± 0.62 (25) | 200.81 ± 23.03 (26) | 9.61 ± 0.43 (51) | 4.15 ± 0.33 (75) | 48.93 ± 0.59 (30) | 45.40 ± 0.54 (30) | 14.50± 0.25 (2) | 17.17 ± 1.85 (12) | 8.50 ± 3.50 (2) |
| 3 | R27, 11km south west of Mamre | S 33° 33’ 56.3” E 018° 23’ 3.2” | 217.89 ± 3.97 (66) | 35.36 ± 5.18 (25) | 21.28 ± 0.45 (25) | 198.61 ± 8.01 (49) | 13.26 ± 0.50 (50) | 3.17 ± 0.24 (136) | 52.24 ± 0.60 (50) | 45.50 ± 0.50 (50) | 12.92 ± 1.28 (13) | 13.00 ± 1.77 (13) | 14.32 ± 0.89 (34) |
| 4 | Kooperfontein, Hopefield district | S 33° 06’ 51.5” E 018° 24’ 01.5” | 213.73 ± 5.07 (69) | 22.37 ± 2.40 (25) | 22.00 ± 0.64 (25) | 228.80 ± 8.55 (49) | 9.26 ± 0.40 (69) | 3.88 ± 0.24 (144) | 47.56 ± 0.58 (50) | 44.92 ± 0.46 (50) | 5.00 ± 1.22 (4) | 6.89 ± 1.54 (19) | 10.60 ± 2.94 (5) |
| 5 | Fanshoek Pass | - | 186 ± 10.4 (4) | - | - | 278 ± 29.07 (4) | 4.25 ± 0.48 (4) | 1.0 ± 0.71 (4) | 86.25 ± 1.65 (4) | 44.25 ± 0.85 (4) | - | - | - |
| 6 | Jakkelsfontein Private Nature Reserve | S 33° 24' 49.8“ E 18° 15' 19.8" | 178.12 ± 7.15 (24) | 27.50 ± 6.10 (5) | 32.50 ± 0.44 (5) | 197.42 ± 12.41 (25) | 10.96 ± 0.73 (25) | 5.35 ± 0.69 (17) | 47.96 ± 0.92 (21) | 41.60 ± 0.97 (22) | 8.11 ± 1.56 (9) | 11.67 ± 0.91 (15) | 10.17 ± 1.77 (12) |
| 7 | Worcester | - | 174.57 ± 4.24 (27) | - | - | 257.68 ± 12.13 (34) | 5.0 ± 0.33 (33) | 1.83 ± 1.17 (6) | 79.57 ± 5.41 (7) | 43.17 ± 3.38 (6) | - | - | - |
| 8 | Clanwilliam | - | 190 ± 20.0 (2) | - | - | 320 ± 35.0 (2) | 6.5 ± 3.5 (2) | 2.5 ± 2.5 (2) | 71.5 ± 16.5 (2) | 45 ± 4.0 (2) | - | - | - |
| 9 | Kraaifontein | - | 160 ± 12.58 (3) | - | - | 350 ± 51.19 (4) | 3.33 ± 0.67 (3) | 7.5 ± 2.06 (4) | 97.25 ± 1.80 (4) | 48.25 ± 3.82 (4) | - | - | - |
| 10 | Lamberts Bay | - | 155 (1) | - | - | 395 (1) | 5 (1) | 0 (1) | 73 (1) | - | - | - | - |
| 11 | Langebaan | S 33° 03' 52.6“ E 18° 04' 49.6" | 155.62 ± 3.0 (4) | - | - | 116.87 ± 18.91 (4) | 6.5 ± 0.65 (4) | 5.74 ± 0.81 (4) | 42.78 ± 2.10 (4) | 42.29 ± 1.93 (4) | - | - | - |
| 12 | Dekriet, 13km west of Albertina | S 34° 11’ 27.5” E 021° 27’ 34.5” | 143.94 ± 4.79 (39) | 22.75 ± 4.37 (4) | 30.25 ± 0.48 (4) | 231.87 ± 21.28 (15) | 4.74 ± 0.34 (34) | 1.47 ± 1.02 (19) | 24.67 ± 0.75 (20) | 41.1 ± 0.64 (20) | 0.25 ± 0.25 (4) | 12.15 ± 0.94 (26) | 11.67 ± 2.17 (6) |
| 13 | Scarborough | S 34° 11’ 35.7” E 018° 23’ 0.6” | 142.17 ± 3.97 (57) | 3.99 ± 0.23 (15) | 21.87 ± 0.66 (15) | 112.63 ± 3.14 (35) | 5.86 ± 0.22 (35) | 2.91 ± 0.48 (35) | 32.69 ± 0.83 (35) | 38.29 ± 1.09 (35) | 3.89 ± 1.22 (9) | 6.88 ± 1.11 (16) | 8.21 ± 0.97 (14) |
| 14 | Victoriasdaal farm, Riversdale | S 34° 20’ 50.1” E 021° 14’ 57.0” | 128.11 ± 1.27 (161) | 14.34 ± 0.71 (50) | 23.16 ± 0.67 (50) | 138.14 ± (100) 3.16 | 7.44 ± 0.25 (140) | 1.50 ± 0.17 (272) | 26.39 ± 0.20 (100) | 27.64 ± 0.19 (100) | 11.30 ± 0.82 (10) | 18.71 ± 1.10 (14) | 18.55 ± 0.75 (21) |
| 15 | Still Bay | S 34° 22’ 43.8” E 021° 24’ 40.6” | 126.20 ± 4.78 (28) | 15.42 ± 2.37 (6) | 30.25 ± 1.03 (4) | 168.11 ± 20.15 (9) | 4.89 ± 0.46 (29) | 0.55 ± 0.64 (31) | 27.00 ± 1.07 (9) | 31.56 ± 0.65 (9) | 3.5 ± 3.5 (2) | 14 ± 0.98 (14) | 5.00 ± 5.00 (2) |
| 16 | Red Hill Road, Simon’s Town | S 34° 11’ 06.2” E 018° 24’ 34.5” | 112.31 ± 1.85 (123) | 23.33 ± 4.41 (3) | 25.67 ± 2.85 (3) | 91.34 ± 2.87 (74) | 3.32 ± 0.15 (74) | 1.37 ± 0.28 (103) | 29.66 ± 0.53 (32) | 36.44 ± 0.54 (32) | 2.20 ± 0.93 (10) | 6.50 ± 0.76 (32) | 5.25 ± 1.29 (8) |
| 17 | Fish Hoek | S 34° 8' 39.7" E 18° 24' 0.03" | 100.28 ± 5.42 (16) | - | - | 343.33 ± 36.09 (3) | 5 ± 1.15 (3) | - | - | - | - | - | - |
| 18 | Jacobs Bay | S 32° 59' 19.9" E 17° 53' 55.2" | 89.49 ± 5.00 (14) | 30.25 ± 4.59 (4) | 29.38 ± 0.85 (4) | 90.25 ± 6.53 (12) | 5.89 ± 0.53 (19) | 1.53 0.41 (14) | 29.31 ± 0.68 (15) | 40.31 ± 0.70 (16) | 0.00 ± 0.00 (9) | 13.69 ± 1.44 (13) | 10.63 ± 1.95 (8) |
