# Supplementary material for: Geographic variation of reproductive traits and competition for pollinators in a bird‐pollinated plant
Source: Ecol Evol. 2019 Aug 20;9(18):10122–34. doi: 10.1002/ece3.5457 (PMC6816071; doi:10.1002/ece3.5457)
Supplement: Supplementary file 3 [file ECE3-9-10122-s003.docx]

**Table S2** Details of pollinator observations showing the total numbers of hours spent observing in each population and over how many days and years those observations were taken. Localities numbered according to Table S1.

| Locality | *Babiana ringens* | Other bird pollinated species | Malachite visitation rate (Visits/plant/h) | Total visitation rate (Visits/plant/h) | Approximate observation area (m²) | Observation period (mins) | Observation  days | Years observed across |
| --- | --- | --- | --- | --- | --- | --- | --- | --- |
|  |  |  |  |  |  |  |  |  |
| 1 | *Babiana ringens* |  | 0.143 (1) | 0.143 (1) | 125 | 180 | 1 | 1 |
| 2 | *Babiana ringens* |  | 0.071 ± 0.09 (3) | 0.071 ± 0.09 (3) | 65 | 270 | 3 | 2 |
| 3 | *Babiana ringens* |  | 0.04 ± 0.10 (4) | 0.04 ± 0.10 (4) | 24 | 510 | 5 | 2 |
| 4 | *Babiana ringens* |  | 0.109 ± 0.11 (4) | 0.109 ± 0.11 (4) | 45 | 420 | 4 | 2 |
| 6 | *Babiana ringens* |  | 0.178 (1) | 0.178 (1) | 100 | 300 | 2 | 1 |
|  |  | *Salvia africana-lutea* | 0.123 (1) | 0.123 (1) | 100 | 300 | 2 | 1 |
| 12 | *Babiana ringens* |  | 0 (3) | 0 (3) | 30 | 200 | 4 | 3 |
|  |  | *Erica versicolor* | 0.256 (1) | 0.41 (1) | 50 | 90 | 1 | 1 |
| 13 | *Babiana ringens* |  | 0.0947 ± 0.04 (4) | 0.0978 ± 0.04 (4) | 150 | 410 | 4 | 2 |
|  |  | *Mimetes cucullatus* | 0.3 (1) | 1.2 (1) | 20 | 120 | 1 | 1 |
|  |  | *Erica plukenetii* | 0 (1) | 0.1 (1) | 30 | 120 | 1 | 1 |
|  |  | *Leucospermum conocarpodendron* | 0 (1) | 4 (1) | 1 | 120 | 1 | 1 |
| 14 | *Babiana ringens* |  | 0 (4) | 0.001 ± 0.01 (4) | 200 | 720 | 4 | 2 |
|  |  | *Leucospermum praecox* | 0.0533 (1) | 0.133 (1) | 40 | 180 | 1 | 1 |
| 15 | *Babiana ringens* |  | 0 (2) | 0 (2) | 70 | 150 | 1 | 1 |
| 16 | *Babiana ringens* |  | 0 (1) | 0 (1) | 200 | 180 | 2 | 2 |
|  |  | *Mimetes cucullatus* | 0.219 (1) | 0.406 (1) | 9 | 240 | 1 | 1 |
| 18 | *Babiana ringens* |  | 0 (2) | 0 (2) | 80 | 310 | 1 | 1 |
|  |  | *Septulina glauca* | 0 (1) | 0.258 (1) | 15 | 310 | 1 | 1 |
